# Assessing project approval procedures as formalised forms of public participation

**DOI:** 10.1007/s10202-012-0124-3

**Published:** 2012-11-27

**Authors:** Michael Zschiesche

**Affiliations:** Independent Institute for Environmental Issues (UfU), Berlin, Germany

## Abstract

Formalised public participation in project approval procedures is rarely addressed in technology assessment. Empirical data about public participation processes are taken into account even more rarely. This article explores the practice of public participation in infrastructure projects in the Federal Republic of Germany on the basis of empirical data from the period of 1990 to 2010. The author compares the empirical data about participation processes with the targets of the public participation and asks for the reasons for the lack of participation in formalised approval procedures. Furthermore, the author contrasts participation formats used in technology assessment with formalised public participation. In his conclusions, he advocates for a stronger combination of formalised and informal public participation in technology assessment.

## Abstract (de)

In der Technikfolgenabschätzung wird die formelle Öffentlichkeitsbeteiligung in Zulassungsverfahren selten thematisiert. Noch seltener werden empirische Daten zu Öffentlichkeitsbeteiligungsprozessen betrachtet. Dieser Artikel setzt sich mit der Praxis der Öffentlichkeitsbeteiligung in Infrastrukturprojekten in der Bundesrepublik Deutschland anhand empirischer Daten im Zeitraum von 1990 bis 2010 auseinander. Der Autor gleicht die Praxisdaten mit den Zielen der Öffentlichkeitsbeteiligung ab und fragt nach den Gründen für mangelnde Beteiligung in formellen Zulassungsverfahren. Weiterhin stellt der Autor Beteiligungsformate der partizipativen Technikfolgenabschätzung der formellen Öffentlichkeitsbeteiligung gegenüber. In seinen Schlussfolgerungen plädiert er für eine stärkere Kombination von formellen und informellen Beteiligungsverfahren im Bereich der Technikfolgenabschätzung.

## Résumé

La participation publique formalisée dans les procédures d’approbation de projets est rarement adressée dans l’évaluation de la technologie. Les données empiriques concernant les processus de participation publique sont considérées encore plus rarement. Cet article explore la pratique de participation publique dans les projets d’infrastructure en République Fédérale d’Allemagne basée sur les données empiriques de la période de 1990–2010. L’auteur compare les données empiriques sur les processus de participation avec les buts de la participation publique et recherche les raisons concernant le manque de participation dans les procédures formalisés d’approbation. De plus, l’auteur contraste les formats de participations utilisés dans l’évaluation de la technologie avec la participation publique formalisée. Dans ses conclusions, il préconise une combinaison plus forte de participation publique formalisée et informelle dans l’évaluation de la technologie.

## Introduction

Germany is an industrial country. Building and extending industrial facilities as well as building and extending infrastructure are given high political priority, but they also have been bringing numerous opponents into the arena for decades. In Germany, 775 approval procedures for new projects and industrial facilities with formalised public participation with regard to environment protection are carried out every year.^1^ There are basically two types of procedures, the authorisation process according to the Federal Immission Control Act for the approval of industrial facilities and the planning permission procedure for infrastructure projects. The authorisation procedure for industrial facilities covers the building or the extension of lignite- or hard-coal-fired power plants, wind power plants, plants for the production of fertilizers, waste incineration plants, carcass disposal plants, pig and poultry farms, biogas plants, etc.^2^ In North Rhine–Westphalia alone, the federal state with the largest number of industrial facilities in Germany, every year 900–1100 permissions for large or small industrial facilities are granted.^3^

Planning permission procedures are carried out mainly for infrastructure projects. The most relevant fields are road building,^4^ railway construction,^5^ the building of airports,^6^ the setting up of general operation plans^7^ and the building of power lines.^8^ Planning permission procedures can generally be carried out regarding the following projects (Schlacke et al. 2010:180f):

- Construction of federal highways and significant changes thereof
- Projects of the major German railway company Bahn AG, especially the construction of railways or major changes of railway facilities
- Construction or significant changes of tram or underground lines
- Stationary waste disposal plants, construction and operation of waste disposal sites and significant changes thereof
- Construction or significant changes of magnetic levitation systems
- Construction of airports or airstrips
- Construction of telegraphy lines
- Construction or extension of federal water ways
- Extension of waterways, maintenance of waterways and bodies of water, construction of dams and significant changes thereof
- Setting-up of general operation plans
- Construction and operation of a facility for the storage and final disposal of radioactive waste and the significant change thereof
- Power lines (high voltage overhead power lines)
- Natural gas pipelines

The two types of project approval procedures have much in common. In both cases, the approval decisions are adopted in the exercise of circumscribed powers and have a concentration effect. This means that all aspects of the projects were examined by different administration departments (such as the nature conservation authority, the water authority and the building authority) during the approval process. They also have a toleration effect. After the approval was granted, any demands to omit the project or to remove or modify the facilities are ruled out. The differences between the two types of project approval procedures are, for example, that an authorisation process according to the Federal Immission Control Act has to be completed after seven resp. 3 months, whereas there is no strict time limit for planning permission procedures. They merely have to be completed “in a timely manner”. In planning permission procedures, the authorities regularly examine the necessity of the project. This is not done in case of an authorisation process according to the Federal Immission Control Act.

## Formalised procedures and difficulties of the public participation in project approval procedures

Basically, public participation in the two described approval procedures takes place following similar principles and procedures. But there are some differences. Public participation in planning permission procedures is governed by the regulations in the Administrative Procedures Act (VwVfG).^9^ If special laws on the building of infrastructure have further-reaching regulations for public participation, these have priority over the more general regulations of the VwVfG. Public participation according to the Federal Immission Control Act is governed by the 9th Federal Immission Control Ordinance.

Whereas the planning permission procedure is a matter of discretion of the administration that is carrying out the examination whether the project is needed (“necessity of the project”) for the intended project, the investor in an authorisation process according to the Federal Immission Control Act has a right to the permission, if he meets all legal requirements.^10^ This principle grants the investor, who can take legal action to enforce his right, a strong position in the authorisation procedure. In such an authorisation process according to the Federal Immission Control Act, the general public, too, can take legal action against the project. The applicant in a planning permission procedure on the other hand does not automatically have the right to the permission for his project, but is only entitled to a decision on the discretion of the relevant administration that is free of mistakes.^11^ Whereas, the general public can enforce a legal examination of the decision in a planning permission procedure, on the one hand, neighbours, whose rights are affected by the project, can stand up against the planning permission in court. On the other hand, recognised environmental organisations have the right to challenge a planning permission in court if provisions of environmental law are affected.^12^

### The course of public participation and significant restrictions of formal participation

The major elements of public participation in planning permission procedures as well as authorisation processes according to the Federal Immission Control Act are: announcement of the project, putting the planning documents on display, objection phase and subsequently a hearing. The following flowchart shows the elements of a planning permission procedure from the point of view of public participation (Tables 1, 2 and 3).

**Table 1:** Schematic course of a planning permission procedure from the viewpoint of the general public (Zschiesche 2008:51)

	Previous steps of planning (regional planning procedure, definition of routes and lines), if applicable with public participation	
	↓	
	Application of the project developer	
	↓	
	Examination of the planning documents for completeness by the administration responsible for the planning permission procedure or the hearing	
	↓	
	Other relevant administration and public agencies are asked for their comments	
	↓	
	Public announcement	
	↓	
	Public display of planning documents	
↓		↓
End of the participation of the general public, in there are no objections		Objections from affected persons or recognised environmental organisations
		↓
	If there are objections, it is within the discretion of the administration responsible for the planning permission procedure to call a hearing	
	↓	
	Further examination by the administration	
	↓	
	Decision on planning permission	
↓		↓
End of planning permission procedure		Legal action

**Table 2:** Proportion of planning permission procedures against planning approval procedures in railway construction in Germany in the years 1999–2004

Federal railway agency branch	Number of projects	Planning permission procedures	Planning approval procedures
Berlin	391	15	376
Dresden	242	16	226
Erfurt	6	3	3
Halle	79	8	71
Hamburg/Schwerin	410	48	362
Hannover	6	6	0
Nurnberg	42	4	38
Frankfurt	0	0	0
Total	1176	100	1076

See report of the German Federal Government on the Transport Infrastructure Planning Acceleration Law, Bundestag printed paper 15/2311 of 2.1.2004 p. 8

**Table 3:** Ratio of permission procedures presented to the public to permission procedures with actual public participation in selected federal states between 2002 and 2009

Federal state/year	Saxony		Thuringia		North Rhine Westphalia		Total A	Total B	Ratio B to A (%)
	A	B	A	B	A	B			
2002	21	8	9	7	64	18	94	33	35.1
2003	20	10	18	2	66	15	94	27	28.7
2004	22	11	16	6	54	11	92	28	30.4
2005	21	12	14	5	60	19	95	36	37.9
2006	23	19	23	7	67	10	113	36	31.9
2007	9	1	5	1	86	24	100	26	26.0
2008	_	_	13	3	118	22	131	25	19.1
2009	_	_	_	_	110	23	110	23	20.9
Total	116	61	98	31	625	142			
Ratio B to A (%)	52.6	31.6	22.7						
Total				829	234	28.2			

The data are based on written information from state ministries resp. state environment agencies in North Rhine-Westphalia, Saxony and Thuringia of 2010 collected by the author

This does not include the comments by acknowledged environmental organisations that are also objecting to authorisation procedures according to the Federal Immission Control Act (BImSchG)

A = Number of planning procedures presented to the public

B = Number of these procedures in which citizens commented or objected

The formal public participation procedures in Germany are suffering from the fact that they are not participation in the decision process, but merely participation by information. Additionally, the existing opportunities for public participation were diminished considerably by the numerous so-called acceleration laws adopted during the unification process in 1990 (Ekardt 2010). Consultative elements or dialogue based set-ups are missing in formalised participation procedures as a rule, which is degrading public participation to a pre-litigation step of a planning procedure. Aims and roles of public participation as conciliation and integration, the search for conflict solutions or the control of the administration have no correspondence in the set-up of formalised participation processes. Additionally, there is criticism that within the logic of formalised participation processes, the participation happens too late; that the time limits, the ways how information can be obtained and the procedure of the discussion meeting as well as the feedback of the results of the participation process are not organised in a fair way from the objectors point of view; that there is too little flexibility to adjust the process to the respective project; and that the administration responsible for the permission procedure has conflicting interests when organising the practical process, for example, the discussion meeting.^13^

### Results of empirical research into the formalised participation of the general public in environment-relevant approval procedures in Germany in the years 1990–2010

Even though environment-relevant approval procedures are of high environment and socio-political importance, systematic research into the actual participation in formalised approval procedures was carried out only marginally in Germany over the last 20 years (Wende 2001; UfU 1993, 2002). The public debate on the effectiveness of formalised participation procedures in Germany is conducted without knowledge of the concrete practice of environment-relevant approval procedures. Even though it is important and correct to discuss and develop new formats and designs of participation and to address the question about how to combine formalised participation procedures with informal formats of participation, the current experience with formalised participation procedures should not be ignored (see Dialogics 2011; Bertelsmann-Stiftung 2011). To ensure the necessary blending of formalised participation procedures and informal formats of participation, it is important to research and analyse the practise of the formalised participation as it is now. The relevant questions in this context are how many permission procedures with public participation are taking place, what are the topics objections and comments from citizens or recognised environmental organisations in the context of technology-related permission procedures, and what effects the objections and comments have on the permission process.

Empirical data on authorisations granted according to the Federal Immission Control Act suggest that only 10 % of all authorisation procedures according to the Federal Immission Control Act are presented to the general public. The remaining authorisation processes are taking place strictly with the administration (UfU 2005). Furthermore, the number of projects presented to the public is decreasing, because German legislature is working towards listing an increasing number of projects in “column 2” of the relevant decree, consisting of projects with lesser environment relevance on the one hand and less large industrial facilities are being planned in Germany on the other hand (German Advisory Council on the Environment 2002:106).

Amongst the planning permission procedures, there is a larger proportion of projects with public participation, as the conditions for ruling out public participation are stricter than in the Federal Immission Control Act. A simpler planning approval procedure instead of a public planning permission procedure can take place, if rights of others are not affected or those affected *have agreed to the encroachment on their property or another right in written form.*^14^ The proportion of planning permission procedures with public participation to planning approval procedures carried out within the administration varies strongly depending on sector and federal state. There are no empirical data for some sectors in which planning permission procedures take place. Thus, only certain sectors can be presented here. In the sector of railway construction, the following data exist.

The table shows that in the sector of railway construction, the public display of project documents is as rare as in procedures according to the Federal Immission Control Act. In road construction, public participation differs from federal state to federal state. In the state of Saxony, 216 public planning permission procedures and 205 planning approval procedures took place between 1990 and 2002.^15^ In the sector of road construction in Rhineland-Palatinate, there were 295 planning permission procedures and only 28 administration-internal planning approval procedures between 1994 and 2001 (Ahlborn 2006:303). In Thuringia, however, the proportion of planning permission procedures to planning approval procedures between 1992 and 2000 was 123–381. Generally, there is the tendency that in the Eastern states more road construction projects are approved within the administration only than in the Western states.

A general estimate on the procedure type planning permission/approval with regard to how many projects are presented to the public and how many are decided within the administration cannot be given due to the lack of empirical data. But the number of projects presented to the public should clearly exceed the 10 % that are reached in procedures on the authorisation of industrial facilities.

The next relevant question is how many of the projects presented to the public (authorisation procedures for industrial facilities and planning permission procedures) were met with interest by the public, that is, were public participation really happened. There are the following data regarding big industrial developments:

These figures show that on average just nearly one-third of all authorisation procedures for industrial facilities displayed to the public are in fact met with public interest and the other 71.8 % take place without participation of the public even though participation would have been possible. If we take not only the procedures covered by column 1 of the relevant decree under die Federal Immission Control Act listing procedures that have to be presented to the public, but all permission procedures under the Federal Immission Control Act, actual public participation took place only in 1.85 % of all cases in the state of North Rhine-Westphalia, the state with the largest number of industrial facilities in Germany. As far as planning permission procedures are concerned, the rate of actual public participation in procedures presented to the public is higher, because in addition to neighbours, affected and general members of the public who comment or object, acknowledged environmental organisations have been taking part in such procedures for many years. A survey done by the German Council for Land Stewardship shows that only 10–15 % of all planning permission procedures are not covered by acknowledged environmental organisations. Therefore, we can assume that the quota of projects with actual public participation in the field of planning permission procedures is 80–20 in favour of the procedures with actual participation public. That means that four out of five planning permission procedures take place with active public participation.

The next relevant question is what kinds of comments are given by the objectors and what kind of effects the objections have. No systematic research has been done into this issue in Germany, there is merely some evidence. The state government of Brandenburg answered a minor interpellation in 2002: *“Most of the objections in procedures conducted by the agencies for immission control and the state environment agency relate to concerns due to the immediate neighbourhood to the projects seeking permission. Very personal concerns play a major role in the objections, such as health issues, loss of value of real estate* etc*. The objections are as diverse as the planned projects and the local conditions. There are three issues that are of utmost relevance for all facilities listed in the 4th Federal Immission Control Ordinance: noise, dust (ingredients of the dust) and odours”*.^16^ A study on the practice of environment impact assessment of 2001 did research into the effects of public participation, too. In his empirical analysis on 120 approval procedures, the author asked explicitly about the correlation between the number of objections and the extent of local and environment protection relevant modifications resp. the extent of general project modifications. He concludes that *“the bigger the number of objections by the public and the §29 (acknowledged environment) organisations,*^17^*the larger is the extent of spatial project modifications and environment protection relevant variants as well as technological modification of general kind (technological, spatial and renunciation of parts of the project) included in planning decisions”* (Wende 2001:171). The author concedes, however, that even though this correlation is deducible, the modifications due to objections by the public *“are of very little weight in the project modifications”* (Wende 2001:171). Further relevant research on the influence of objections and comments on environment-relevant approval procedures is not on record in Germany.

## Comparison between procedures of participative technology assessment and formalised participation procedures

Despite the evidently incomplete empirical research, the previous deliberations show that public participation in formalised environment-relevant approval procedures in Germany in the years between 1990 and 2010 does not comply with the requirements of modern public participation aims.^18^ Most of the experts in this field share this view (see, for example, Dialogics 2011; Bertelsmann-Stiftung 2011; Politische Ökologie 2012 and others). Solutions proposed for a better public participation, however, differ considerably. While this is not the place to rehash the expert discussion, there are two demands and requirements and conceptual approaches that are mentioned by participation researchers and politics alike. Firstly, public participation should happen earlier. Secondly, a combination of formalised and informal forms of participation is to guarantee better results of public participation. With these conceptual approaches, the discussion moves in direction of the aims, which the participative technology assessment has been formulating for years (Grunwald 2010:128). Neither of the approaches is new. The postulate of earliness (Fisahn 2004:136–140) has been reprehensibly neglected by legislation in Germany. The combination of formalised and informal forms of participation is not a new demand either, as the implementation of elements of environment mediation in public participation was addressed in the discussion about the establishment of an environmental code as early as in the 1990s (Zilleßen 2000). What is new is the necessity to give public participation in environment-relevant permission procedures a higher priority, thus giving participation a higher priority in society in general. Assuming that these two proposals for readjusting public participation are reasonable and spot-on, we should discuss which new requirements public participation processes have to meet, if the two proposals are carried through.

It is highly desirable for public participation to take place as early as possible, when all or at least a number of options are still possible, but it requires forms of participation that can handle a whole bundle of options. German legislation established early public participation in grid expansion within the energy turnaround announced hastily in early summer 2011.^19^ But the usual set up of participation was maintained, with elements of early public participation according to the Grid Expansion Acceleration Act being public announcement, putting of planning documents on public display as well as the submission of comments and objections. But early participation poses very different challenges for citizens and environmental organisations. If there are options for different routes that can be taken, there has to be the opportunity to study the different variants. A one-off information session as planned at present,^20^ does not meet the requirements of such a situation. At this stage, approved informal methods that are being used regularly in participative technology assessment as the citizens conference (Joss 2003:15–35), the round table (Fischer and Hänisch 1999) or the dialogue process^21^ could be used that would safeguard that public participation takes place in a way that fits in with the early step of the planning procedure. This way the combination of formalised and informal participation procedures, the second conceptual approach in the current debate on public participation after Stuttgart, would take place as well. But this example shows as well, that it is not enough to make a law comprising early public participation. Corresponding personal and financial resources are necessary to implement the sophisticated methods of the participative technology assessment (Grunwald 2010:80f.).

## Summary and conclusions regarding formalised procedures of public participation in Germany

Public participation in authorisation processes according to the Federal Immission Control Act as well as in planning permission procedures for infrastructure projects are examples for public participation in specific technology assessment. The general public in Germany has the opportunity to take part in 775 ± 150 procedures of that kind every year. Empirical data regarding the frequency of actual public participation show that only one in three authorisation processes according to the Federal Immission Control Act is met by public interest. In the field of planning permission procedures, a significantly higher number of projects displayed to the public are met with public interest due to the participation of acknowledged environmental organisations. Here, four of five projects enjoy actual public participation. There is no solid data regarding the influence of the public on any of these procedures, as no evaluable empirical studies on this issue have been carried out in Germany. Still, there is no sensible combination of informal and formalised public participation. This reduces the likelihood of success of formalised public participation. On the other hand, informal participation procedures are facing a number of major challenges, too, such as the acceptance of the results of informal participation processes (Grunwald 2010:80f.). Participation methods used in participative technology assessment as citizens’ conferences, round tables or dialogue processes have not been implemented in formalised public participation in Germany yet, but they can complement them in a meaningful way. Hereto legislation has to realign public participation fundamentally. Apart from public participation at a much earlier stage of a planning process material means have to be provided to enable the often complicated technical discourse between the public, authorities and investors.

## Footnotes

1 There is no figure of permissions granted in Germany. Therefore, a calculated estimate for five model regions for 2005 done within a research project for the Federal Environment Agency is the most reliable source. It results in a figure of 772 ± 150 EIAs as arithmetic average of three pari passu approaches—As public participation is compulsory in EIA, the figures for EIA and procedures with public participation are identical, see Federal Environment Agency (2009:29).

2 See 4th Federal Immission Control Ordinance (4.BImSchV).

3 See Information system on substances and facilities of North Rhine-Westphalia, analysis of data of 31 December 2009, ministry for environment, nature protection, agriculture and consumer protection of North Rhine-Westphalia, June 2010: http://www.umwelt.nrw.de/umwelt/pdf/ISA_jahresbericht_2009.pdf (22 March 2012).

4 Between 17 December 2006 and mid October 2010, 703 planning permission procedures took place for federal road construction projects in Germany, see answer to minor interpellation, Bundestag printed paper 17/3331 of 19 October 2010.

5 Between 17 December 2006 and mid October 2010, 384 planning permission procedures were carried for railway projects, see answer to minor interpellation, Bundestag printed paper 17/3331 of 19 October 2010.

6 In airport construction, it is not so much the number of projects that impress, but the extent of the projects and the related conflicts, outstanding examples of which in the last 10 years, have been the extension of the airport in Frankfurt and the construction of the new airport in Berlin-Schönefeld.

7 Mining is relevant mostly because of its severe affects in the environment. Lignite mining, as at “Garzweiler II” in North Rhine-Westphalia or “Horno” in Brandenburg, became focal points of public debate known throughout Germany.

8 Almost all sections of planned power lines are highly controversial in the public. German Federal Government adopted a Grid Expansion Acceleration Act (NABEG) in summer 2011 with improved provisions for public participation.

9 See Administrative Procedures Act (VwVfG) §§ 73 f.

10 See § 6, Federal Immission Control Act (BImSchG), the permission according to immission control regulations has to be granted, if the operator has fulfilled the basic obligations mentioned in § 5 BImSchG and no other regulation under public law bar the construction or the operation of the facility.

11 Case law assumes that, if the project has no negative effects on the general public or third parties or such effects can be prevented by additional requirements, there is a de facto right to a planning permission due to the reduction in the margin of discretion to zero (see Kopp, § 72 Rdnr. 41).

12 These are based on the infringement of federal nature protection regulations (§ 64 Abs. 1 BNatSchG) resp. state nature protection regulation on the one hand and infringements relevant according to the Environmental Appeals Act (§ 2 Abs. 1 UmwRG, due to which general infringements of environmental law can be rebuked).

13 See statement by Öko-Institut, Deutsche Umwelthilfe and Unabhängiges Institut für Umweltfragen (UfU) e.V. on the draft of the Environmental Code at www.umweltgesetzbuch.org (22 March 2012).

14 § 74 VwVfG Abs. 6 Administrative Procedures Act as announced on 23 January 2003 (BGBl. I p. 102), last amended by article 2 paragraph 1 of the Act on 14 August 2009 (BGBl. I p. 2827) as well as respective sectoral laws.

15 See state parliament of Saxony, printed paper 3/8648 of 04 September 2002, p. 2.

16 See Brandenburg state parliament printed paper 3/4931.

17 Till the amendment of the BNatSchG in 2002, the acknowledged environmental organisations were referred to as § 29-organisations, the respective paragraph on the participation of environmental organisations being § 29 BNatschG war. The amendment of BNatSchG changed the numbering of the paragraphs. The participation of environmental organisations now follows § 58 BNatschG.

18 The target is derived from the Aarhus Convention as well as the Directive on Public Participation (EU/2003/35) in: UfU 2011:37.

19 See § 9 Grid Expansion Acceleration Act (NABEG) transmission network of 28 July 2011, BGBl I p. 1690.

20 See § 10 NABEG.

21 IFOK, Pluspunkt—Dialogverfahren bei Infrastrukturprojekten, p. 8: http://www.ifok.de/einblicke/pluspunkt/ (12 February 2012) and in: UfU 2011.
